# Remarks on Follicular Enteritis, Occurring as a Complication of Continued Fever

**Published:** 1847-12

**Authors:** 


### BUFFALO MEDICAL JOURNAL

AND

### MONTHLY REVIEW.

**VOL. 3.** DECEMBER, 1847. **NO. 7**

### ORIGINAL COMMUNICATIONS.

ART. I.—*Remarks on Follicular Enteritis, occurring as a complication of continued Fever.* By the EDITOR.

The morbid conditions of the intestinal mucus follicles, more especially of those known as the aggregated follicles, or the glands of Peyer, considered as an element of continued fever, have of late years occupied not a small share of medical literature. Our views upon several interesting questions of Pyretology, at the present time, must be influenced considerably by our estimation of the nature and extent of the pathological relations of these lesions. We purpose to offer a few remarks, first, on follicular enteritis as a complication of continued fever in general; and, next, as constituting a distinguishing characteristic of a particular species, or variety of continued fever.

According to Cruveilhier, these lesions were first described by *Prost*, who published his observations in 1804. They were next treated of, and considered as constituting a special malady, by *Petit and Serres*, French Pathologists. Broussais recognized them, to some extent, in the *gastro-enterite*, which, in his view, comprised the essential pathology of all fevers. Afterward, *Brettonneau* described them as a kind of internal eruptive affection, and gave to the disease the name of *Dothinenterie*, signifying intestinal pustule. To Louis, however, is due the credit of having investigated, more carefully than any previous observer, the various characters which belong to them intrinsically, and the relations which they sustain to the fevers of Paris. The interest and importance which of late years have attached to them, may be dated from the publication of the admirable work on fever by the distinguished pathologist just named.

This work embraced the results of an analysis and numerical comparison of a large collection of observations, gathered chiefly at the hospital *La Charite*, contrasted in all important points, both of symptoms and lesions, with an equally large number of cases of other acute diseases. One of the most striking of these results was the fact, that, as respects pathological changes found after death, more or less disease of the agminated follicles of the ileum, and of the mesenteric ganglions, was invariably found in the dissection of the fever subjects, while similar forms of disease, of the same parts, were found in none of the subjects dead with other acute diseases. Moreover, this was the sole appreciable, local complication which was found in *all* the cases, other lesions existing, each in a greater or less proportion of cases, but none of them constant, From the constancy of its connection in the cases analyzed by him, M. Louis deduced the conclusion, that it is an invariable, integral element of continued fever; and he distinguished it as the "anatomical characteristic" of the *typhoid affection*, employing this latter expression as synonymous with the continued fever of most nosologists.

The follicular lesions described by Louis are not uniform. He divides them into two classes. In the first class, the patches, (which are scarcely, if at all visible, with the naked eye in a state of health,) are rendered distinct by a greater development of the follicles. They appear to be simply hypertrophied in some cases. When considerably enlarged, so as to be separately prominent, they may present a granulated appearance, resembling small pustules; hence, the affection was called, but incorrectly, by Brettonneau, *Dothinenterie.*

In this class of lesions the mucus membrane is thickened and softened, and in a large proportion of cases ulcerated. The ulcerations vary in size, number, form, depth, and other characters. The softening may, or may not be accompanied with redness; and the mucus membrane in the spaces intervening between the patches, may, or may not participate in the follicular disease.

In the second class of lesions the patches are *hard*, instead of being softened. and the seat of the disease seems to be the *submucus tissue*. This tissue wholly, or nearly throughout the patches, becomes converted into a homogeneous substance, having no appearance of organization, of a pale rose or yellowish color, presenting a dry, shining appearance when cut, either friable or having some consistence, and from two to three lines in thickness. This substance, it was apparent, was not secreted upon the surface of the submucus tissue, but *into its substance*. This form of the affection was found in one-third of the patients: 13 out of 49. This substance is identical with the "typhous material" of the German pathologist, regarded by them as characteristic of a particular form of fever, which they distinguish as "abdominal typhus."

Louis does not undertake to decide that either of the lesions, viz., the mucus and submucus, is invariably primitive with regard to the other, when they are found together, as they not infrequently are. He inclines to the opinion that in some instances they commence simultaneously, but from the fact that cases occur in which the submucus deposit exists in abundance, while the mucus tissue remains unaltered, he thinks it fairly deducible that the former *may* take precedence of the latter.

The specific character of the follicular affection is derived mainly from the sub-mucus deposit, and that, in this respect, the affection is, to a certain extent, peculiar to fever, is evidenced by the fact, that in the greater number of cases in which the mucus membrane is inflamed, irrespective of fever, the sub-mucus tissue does not participate in the disease.

The lesions thus briefly sketched are limited to the last eight feet of the small intestine. In the same cadaver different degrees of disease are manifested in different sections of the intestinal tube, the most intense being invariably in the patches next to the coecum.

Sometimes every gradation of disease is illustrated in a single autopsy, commencing with the most extensive lesions next the coecum, and those of a lesser grade, progressively apparent as the canal is examined upward, toward the duodenum. These facts show that the alterations of the lower portion of the ileum are primitive, and that the disease proceeds in a gradual manner upward, attacking the patches successively. Again, in dissections of persons who died after a protracted duration of fever, the follicles nearest the coecum exhibited a greater progress toward cure than those above, and, progressively, the evidences of restoration diminished, as the examinations were continued upward, showing that in the progress of reestoration, as of disease, the same law holds good.

The question now arises, what pathological relations do these lesions sustain to continued fever?

Louis, and others who have adopted his views, regard these lesions as constituting not the disease, but an essential element of it. Quoting his language they "form an anatomical characteristic (of typhoid fever) as much as tubercles do of Phthisis." In the relation which they sustain to fever, they have also been compared to the eruption in small-pox, and the other exanthemata. Some of the French pathologists, however, appear to regard it in a more important light. Cruveilhier, for example, in his *anatomie pathologie*, describes these lesions under the head of "*acute primitive follicular enteritis*," ond considers them as constituting the disease fully as much as the local affection constitutes the disease in pneumonitis, pleuritis, &c. Continued fever, with this complication, he considers should be ranked among the phlegmasiæ; but with respect to the phlegmasiæ generally, he entertains some peculiar views which serve to qualify his opinion that continued fever is symptomatic of follicular enteritis. He says there are two things involved in follicular enteritis, as in all other phlegmasiæ:—a local condition and a general condition. In the phlegmasiæ *with pain*, it is the local condition which originates, and, for the most part, regulates therapeutical indications. In phlegmasiæ *without pain*, it is the general condition, or, to speak more precisely, the state of the cerebro spinal nervous system, which presides over these indication.

We translate literally some of his remarks on the pathology of the affection, contained in the work already referred to. Having acknowledged that the local affection is proceeded by general febrile symptoms, he says:—

"But the local condition, although consecutive (consecutive in the same manner as pneumonitis and pleuritis), and although latent, in a great number of cases, is not to be despised. Once produced, it elevates itself to the rank of a cause. It seems to regulate by its degrees, by the greater or less rapidity of its march, and by its different phases, the degrees, the march, and the phases of the malady. The ganglionary nervous system, which is distributed so abundantly to the diseased parts, explains, in this instance, as in strangulated hernias, the excessive prostration of the forces, and the part which the central nervous system plays—the torpor, stupor, and delirium. The state of the heart, which I regard, in general, as the thermometer of the state of the ganglionary nervous system, and the state of the muscles, which I regard as the thermometer of the cerebro-spinal system, give in general, sufficiently well, the measure of the gravity of the affection, and, sometimes, also, the susceptibility of the subject." The author premises these remarks by saying "that the oldest observers recognized a certain class of maladies which have an evident point of departure in pain and other local symptoms; and another class, which have no such manifest point of departure. The last are distinguished as fevers. Pathological anatomy reveals the great truth, that there are a great number of inflammations without pain, and without notable local symptoms, but that they re-act on the nervous system, and circulating system, and all the functions, in a manner perhaps more intense than phlegmasiæ with pain. The typhoid state is common to all inflammations without pain, but especially to *acute follicular enteritis*."

We have quoted these views on account of their intrinsic interest, as well as the high quarter whence they emanate. It will be perceived that although Cruveilhier styles the disease *primitive follicular enteritis*, he does not advocate the doctrine that this is really the primitive ailment. He considers it as an effect of a previous general condition (fever) but a secondary cause of certain of the phenomena which go to make up the disease. The peculiarity of his views consists, not in considering all essential fevers symptomatic, but in regarding most local inflammations virtually fevers, i. e., effects of a general morbid condition analogous to the morbid condition preceeding the local complications of fever. The latter idea, although probably not true to the extent just enunciated, is yet not devoid of truth. The proposition contains, in our view, important truth which has been too much overlooked: but our present limits will not permit discussions which would grow out of this train of reflection.

The views of Cruveilhier and Louis, are not, thus, in reality, very far apart. Louis, although he deems the follicular affection a complication, yet is disposed to attribute to it considerable causative agency in the events of the typhoid affection. He states that the affected patches, considered in the aggregate, will form a considerable area of diseased mucus surface; as much as in many cases of erysipelas, when the latter is a serious affection. His own facts, however, show that the severity and extent of the follicular affection bear no constant proportion to the severity of the prominent general symptoms of the fever. The duration of the fever is not dependent on the condition of the patches; for, when death occurs at a late period, the latter frequently exhibit a considerable advance in the processes of restoration; and, on the other hand, ulcerations exist, and perforation of the intestine has frequently been known to occur, during convalescence. There are local abdominal symptoms which have a pretty constant, but by no means an invariable relation to the affection. These are meteorism, obscure tenderness, gurgling on pressure over the right iliac region, diarrhœa with yellow ochre colored discharges. The latter, by Dr. Bright and others, have been considered as furnishing a very certain criterion. But that the condition of the nervous symptom, the prostration of the vital forces, and the general course of the disease are closely connected with, or dependent upon the local affection is far from being established. The pathological explanations of Cruveilhier, although plausible and ingenious, are not sustained, but directly controverted by the facts which have just been cited.

We have thus far spoken of follicular enteritis as if it were a constant, and exclusive complication of continued fever. The degree of its constancy is obviously an important point, with reference to the relations which those lesions sustain to continued fever. Louis declares that he found the complication in all the dissections made by him during the observations upon which his work is based, viz., forty-six in number. He supposed that he had discovered a common bond, linking together all the continued fevers, known under the various titles, ataxic, adynamic, putrid, typhus, &c. His original expectation was, that all these different species, or varieties of fever, were to be embraced under the appellation of the "typhoid affection," notwithstanding this involved the very improbable supposition, that, this constant complication should have escaped the previous observations of pathologists of other countries as well as of France. The co-existence of disease of Peyer's glands had been before noticed, but no one, before Louis, had regarded it as an invariable and peculiar element of fever. In the latter conclusion, the chief novelty of the results of the observations of Louis consisted in so far as this affection is concerned. The publi_ cation of Louis' work induced, at once, pathologists of different countries to direct their attention, with particular interest, to the intestinal tube. Dr. Lombard, of Geneva, who visited Edinburg and Dublin shortly afterward, having previously studied the fevers of Paris, was surprised to find that the fevers in the two former cities, althongh they resembled in most of their characters, the fever of Paris, did not constantly exhibit this complication. In some instances it was present, but in many it was absent. Dr. Shattuck jr., also, shortly afterward published some observations made by him at London, in which this complication was wanting. Accumulating facts in the British Islands have shown, that, in the continued fevers of those countries, it is an occasional, but by no means a constant complication. In this country the observations of Drs. Hale and James Jackson, of Boston, Dr. Bartlett (formerly of Lowell, Mass., now of Lexington, Ky.,) and others, established the fact that it accompanies the common continued fever of New England. Dr. Gerhard, and others, of Philadelphia, found it in the fever endemial in that neighborhood, but absent in an epidemic fever which prevailed at Philadelphia in 1836, appearing to have been imported from Liverpool, England, in emigrant ships.

A very interesting and important question has grown out of the fact that it has sometimes found to be present, and, in other instances absent, by these, and other observers. We shall discuss that question hereafter: limiting now our attention to this fact (which is established sufficiently by the instances mentioned, without citing a greater number) it disproves the conclusion of Louis, drawn from his observations, with respect to the co-existence of follicular enteritis in all cases of continued fever. A larger collection of data shows that his generalization was premature. We may add, that observations in France, since the publication of his work, have discovered cases there, in which this affection is absent. Grisolle, for example, states that he has met with such cases. There can be no doubt, however, that it is more generally present in the fevers of France, than in the fevers of Great Britain. It would appear, also, to be less common in the latter than in the endemial fevers of this country. In so far, then, as continued fever, nosologically speaking, denotes a generic class of diseases, the folliular complication is to be regarded, like other complications, not an integral element of the disease, but an occasional result, or local expression.

The fact that it is not uniformly present, suffices to show that it is not a characteristic; but we may inquire in this connection, does it occur except as a complication of continued fever? Louis examined the intestinal canal in a large number of subjects dead with various acute disease, and in no instance were the glands of Peyer found diseased. They have been found diseased in Scarlatina, Phthisis,Cholera, Remitting fever, in the bodies of children after prolonged irritation from dentition, small pox, &c. But the proportion of cases in which they are diseased in these maladies, offers no comparison with the frequency with which they are diseased in continued fever. Moreover, it is affirmed, that when associated with other maladies than contined fever, the morbid characters which the diseased glands present, are different: in other words, that they are specifically affected in continued fever. Grisolle states that the distinctive characters which belong to the follicular complication, as it occurs in the fevers of Paris, are so obvious, that no one practically conversant with them, can fail to distinguish the disease from these characters alone. We presume (for he leaves us to presume on this point) that the distinctive characters to which he refers, are derived from the presence of the sub-mucus affection, or the deposit of the typhous material. Others have found it not easy to distinguish these characters from those presented in other diseases, especially in Phthisis, and after dentition in children. But the morbid characters arising from the sub-mucus deposit are not present in all cases in which follicular enteritis occurs as a complication of continued fever. Louis found these characters well marked in only one third of his fatal cases. Hence, although, when these characters are present, they may indicate pretty certainly that the patient died with continued fever, yet, when not present, even when the follicles exhibit ulcerations, and other morbid appearances, we cannot positively say that the fatal disease might not have been scarlatina, &c. The law of probabilities, however, would be entitled to have considerable weight in this instance. When we consider the frequent occurrence of follicular disease as a complication of continued fever, and its very unfrequent occurrence in other forms of fever, as well as other maladies, beside fever, we have strong grounds for predicating the former state upon the existence of this disease, whether it present any special characters or not, but of course, still stronger grounds for this conclusion, if these special characters are present.

We may now recur to the question, to which allusion has been made, growing out of the fact that the affection under consideration is not a constant, but an occasional complication. The question is this:—does it, when present, constitute a distinct species, or variety of continued fever? This question leads us to a point just now much mooted among medical controversialists, viz., the identity of Typhus and Typhoid fevers. As we shall not have space to finish the discussion of this interesting topic, in so far as it is involved in our present subject, we will here conclude, and resume our remarks in the next No.

